# Evaluation of Host Serum Protein Biomarkers of Tuberculosis in sub-Saharan Africa

**DOI:** 10.3389/fimmu.2021.639174

**Published:** 2021-02-25

**Authors:** Thomas C. Morris, Clive J. Hoggart, Novel N. Chegou, Martin Kidd, Tolu Oni, Rene Goliath, Katalin A. Wilkinson, Hazel M. Dockrell, Lifted Sichali, Louis Banda, Amelia C. Crampin, Neil French, Gerhard Walzl, Michael Levin, Robert J. Wilkinson, Melissa S. Hamilton

**Affiliations:** ^1^Department of Infectious Disease, Faculty of Medicine, Imperial College London, London, United Kingdom; ^2^Department of Genetics and Genomic Sciences, Icahn School of Medicine at Mount Sinai, New York, NY, United States; ^3^DST-NRF Centre of Excellence for Biomedical Tuberculosis Research, South African Medical Research Council Centre for Tuberculosis Research, Division of Molecular Biology and Human Genetics, Department of Biomedical Sciences, Faculty of Medicine and Health Sciences, Stellenbosch University, Cape Town, South Africa; ^4^Centre for Statistical Consultation, Stellenbosch University, Cape Town, South Africa; ^5^Department of Medicine, Wellcome Centre for Infectious Diseases Research in Africa, Institute of Infectious Disease and Molecular Medicine, University of Cape Town, Cape Town, South Africa; ^6^MRC Epidemiology Unit, University of Cambridge, Cambridge, United Kingdom; ^7^The Francis Crick Institute, London, United Kingdom; ^8^Department of Infection Biology, Faculty of Infectious and Tropical Diseases, London School of Hygiene & Tropical Medicine, London, United Kingdom; ^9^Malawi Epidemiology and Intervention Research Unit, Karonga Prevention Study, Lilongwe, Malawi; ^10^Department of Infectious Disease Epidemiology, Faculty of Epidemiology and Population Health, London School of Hygiene & Tropical Medicine, London, United Kingdom; ^11^Institute of Health and Wellbeing, University of Glasgow, Glasgow, United Kingdom; ^12^Department of Clinical Infection, Microbiology and Immunology, Institute of Infection and Global Health, University of Liverpool, Liverpool, United Kingdom

**Keywords:** serum, protein, biomarker, tuberculosis, diagnosis, HIV, Africa

## Abstract

Accurate and affordable point-of-care diagnostics for tuberculosis (TB) are needed. Host serum protein signatures have been derived for use in primary care settings, however validation of these in secondary care settings is lacking. We evaluated serum protein biomarkers discovered in primary care cohorts from Africa reapplied to patients from secondary care. In this nested case-control study, concentrations of 22 proteins were quantified in sera from 292 patients from Malawi and South Africa who presented predominantly to secondary care. Recruitment was based upon intention of local clinicians to test for TB. The case definition for TB was culture positivity for *Mycobacterium tuberculosis*; and for other diseases (OD) a confirmed alternative diagnosis. Equal numbers of TB and OD patients were selected. Within each group, there were equal numbers with and without HIV and from each site. Patients were split into training and test sets for biosignature discovery. A nine-protein signature to distinguish TB from OD was discovered comprising fibrinogen, alpha-2-macroglobulin, CRP, MMP-9, transthyretin, complement factor H, IFN-gamma, IP-10, and TNF-alpha. This signature had an area under the receiver operating characteristic curve in the training set of 90% (95% CI 86–95%), and, after adjusting the cut-off for increased sensitivity, a sensitivity and specificity in the test set of 92% (95% CI 80–98%) and 71% (95% CI 56–84%), respectively. The best single biomarker was complement factor H [area under the receiver operating characteristic curve 70% (95% CI 64–76%)]. Biosignatures consisting of host serum proteins may function as point-of-care screening tests for TB in African hospitals. Complement factor H is identified as a new biomarker for such signatures.

## Introduction

Tuberculosis (TB) remains a leading cause of death from any infection worldwide. The number of people accessing treatment is increasing each year, but in 2019 there were still an estimated 10 million cases and 1.4 million deaths (1). The region with the highest incidence and fatality rate is Africa, where the prevalence of HIV co-infection in some areas exceeds 50% (1).

The potential for rapid diagnosis of TB in African hospitals has been enhanced by the roll-out of the GeneXpert MTB/RIF test (Xpert, Cepheid, Sunnyvale, California, USA). Xpert is a sputum-based PCR assay with high sensitivity and specificity (2), but has several practical limitations. These include high cost, need for annual overseas calibration, laboratory containment facilities, and continuous electricity. In addition, as a laboratory-based assay, Xpert is not a true point-of-care (POC) test that can deliver a result within a single consultation.

An alternative to pathogen detection is quantification of host-derived biomarkers, such as serum proteins. Serum proteins are generally of higher abundance than pathogen products, are amenable to existing POC technologies such as lateral flow immunoassay (LFA), and have been shown to discriminate between different infections when combined as biosignatures (3–6). In 2016, a cohort study was published by the African European TB Consortium (AE-TBC) in which a seven-protein signature was reported that distinguished pulmonary TB from other respiratory diseases with an area under the receiver operating characteristic (ROC) curve of 91% (7). The study was conducted in primary care clinics across five countries in Africa. Participants presenting with symptoms requiring investigation for TB were recruited. The seven proteins were selected from a shortlist of 22 that had been discovered in pilot studies.

An accurate, cheap, user-friendly POC test for TB for use in secondary care hospital settings in sub-Saharan Africa would also be highly desirable. We therefore retested the signature and all 22 biomarkers from the AE-TBC study in cohorts from a case-control study that recruited adults presenting with features of TB to hospitals in Cape Town, South Africa, and Karonga, Malawi, and a TB clinic in Cape Town (the “ILULU-TB study”) (8). Equal numbers of patients were recruited with and without HIV to both TB and other diseases (OD) groups (8). Recruitment of TB patients at all sites was on the basis of culture positivity. All OD patients were recruited from hospitals. We therefore considered this cohort to be reflective of patients presenting to secondary care. We hypothesised that the seven-protein signature from the AE-TBC study, or a new signature derived from the same 22 proteins, would distinguish TB from OD in patients from the ILULU-TB study, regardless of HIV status, with a similar degree of accuracy as in the AE-TBC study.

## Methods

### ILULU-TB Patient Recruitment and Biobank Sampling

Between 2007 and 2010, 674 adults were recruited to the ILULU-TB study from Cape Town, South Africa, and Karonga District, Malawi. These sites have differing prevalences of ODs such as parasitic infection and differing environmental exposures (urban vs. rural). Details of recruitment have been described previously (8). Briefly, patients in the TB and OD groups were recruited consecutively and based on intention of the local clinician to test for TB. The criterion for inclusion in the TB group was at least one positive culture (sputum or tissue) for *Mycobacterium tuberculosis* (Mtb), which is the WHO gold standard (1). Laboratory identification of Mtb was confirmed by polymerase chain reaction (PCR). All of the TB patients that were enrolled had pulmonary TB. OD patients had an established alternative diagnosis, negative cultures for Mtb and an observed improvement of symptoms after follow-up without TB treatment. In Cape Town, TB patients were recruited from either an outpatient clinic (Khayelitsha site B) or hospital sites (Groote Schuur and GF Jooste), whereas OD patients were all recruited from the hospital sites. In Karonga, both TB and OD patients were recruited from Karonga District Hospital. As healthy controls, adults with latent TB infection (LTBI) were also recruited. LTBI status was defined by positive tuberculin skin tests and in-house interferon-gamma release assays in the absence of TB symptoms (9). Sera were collected from all participants at recruitment and stored at −80°C. All groups had HIV-1 status ascertained.

**Figure 1:**
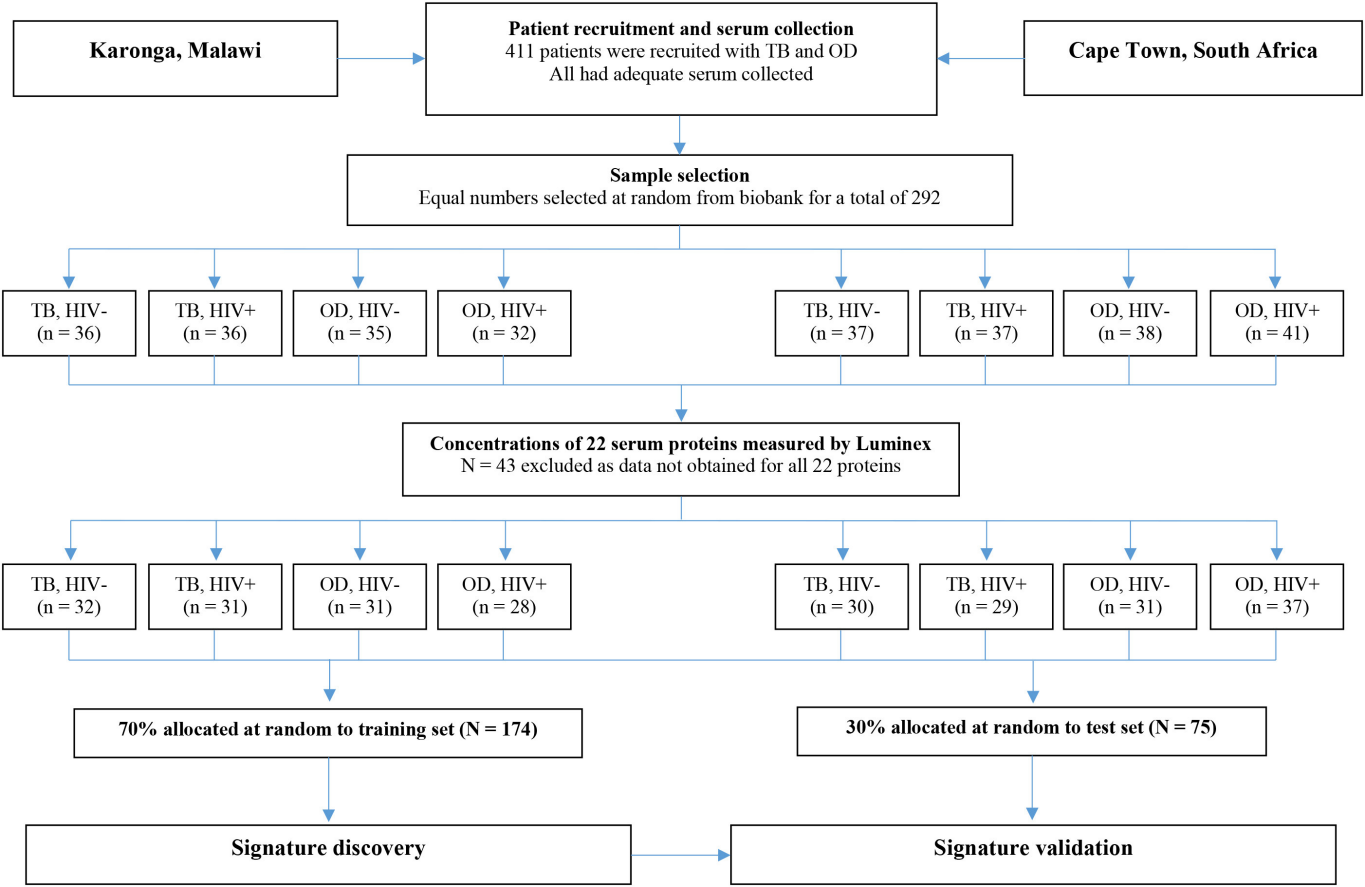
Selection process for inclusion of patients in the biosignature analyses. The flow diagram shows the process from original recruitment to the ILULU-TB study onwards. TB, tuberculosis; OD, other diseases; HIV−, HIV uninfected; HIV+, HIV infected.

For the present study, sera from 438 individuals were selected from the ILULU-TB biobank using random number generation (Microsoft Excel 2013). Equal numbers were selected for each of the TB, OD, and LTBI groups. Within each group, equal numbers were selected with and without HIV, and from each of the two sites (Table 1). The primary aim was to distinguish TB from OD, regardless of HIV status or site. The selection process with regard to the TB and OD patients is illustrated in Figure 1. No sera from the AE-TBC study were re-analysed as part of this study.

**Table 1:** Demographic and clinical features for the 438 participants randomly selected from the ILULU-TB cohort for this study.

	**TB, HIV–**		**TB, HIV+**		**LTBI, HIV–**		**LTBI, HIV+**		**OD, HIV–**		**OD, HIV+**	
	**CPT**	**Karonga**	**CPT**	**Karonga**	**CPT**	**Karonga**	**CPT**	**Karonga**	**CPT**	**Karonga**	**CPT**	**Karonga**
Number	37	36	37	36	40	33	37	36	38	35	41	32
Age (IQR)	32·5 (26·5–41·9)	36 (25·5–53·4)	33·8 (29–37·9)	33·2 (28–39·7)	20·7 (19·3–23·4)	39 (32·4–51·4)	31·2 (27·9–35·1)	44·5 (35·5–49)	41·2 (29·2–51)	43·2 (27·5–53·6)	33·6 (28·6–36·7)	33·3 (29·4–41·2)
Male (*n* (%))	25 (69·4)	19 (52·8)	16 (43·2)	19 (52·8)	16 (40)	15 (45·5)	10 (27)	9 (25)	17 (44·7)	11 (31·4)	16 (40)	11 (34·4)
CD4+count (IQR)	n/a	n/a	170 (69–293)	168 (45–276)^a^	n/a	n/a	345 (231–523)	312 (246–421)	n/a	n/a	183 (95–272)	182 (107–229)
On ART (*n* (%))	n/a	n/a	2 (5·4)	8 (22·2)	n/a	n/a	1 (2·7)	0 (0)	n/a	n/a	18 (45)	12 (37·5)
BMI (IQR)	20·4 (18·4–23·6)^b^	18·4 (16·6–19·3)	20·9 (18·6–23·4)	18·8 (18–20·8)	23·6 (21·5–28·7)	22·7 (20·6–23·7)	24·3 (20·9–27·9)	21·6 (18·7–23·7)	22·4 (20·1–23·8)^c^	20·8 (19·6–23)	21·2 (19·8–23·9)^d^	19·6 (18·1–21·5)

*These are shown by study site for each of the six clinical groups: active TB (TB), healthy controls with latent TB (LTBI), and unwell patients with other diseases (OD) who initially had TB in their differential diagnosis. The definition of a TB case was culture positivity for Mycobacterium tuberculosis (Mtb). The definition of an OD case was a confirmed other diagnosis plus exclusion of TB. HIV+, HIV infected; HIV–, HIV uninfected; CPT, Cape Town; IQR, interquartile range; ART, antiretroviral therapy; BMI, body mass index; n/a, not applicable. Numbers of missing values were <5 except where indicated by letters a–d (a = 6, b = 5, c = 19, d = 20)*.

### Immunoassays

Luminex assays were used as per the AE-TBC study for quantification of interleukin-1 receptor antagonist (IL-1RA), transforming growth factor alpha (TGF-alpha), interferon gamma (IFN-gamma), IFN-gamma-inducible protein 10 (IP-10), tumour necrosis factor alpha (TNF-alpha), IFN-alpha-2, vascular endothelial growth factor (VEGF), matrix metalloproteinase-2 (MMP-2), MMP-9, apolipoprotein A-I (apo-AI), apo-CIII, transthyretin, complement factor H (complement FH) (Merck Millipore, Billerica, Massachusetts, USA); and C-reactive protein (CRP), serum amyloid A (SAA), serum amyloid P (SAP), fibrinogen, ferritin, tissue plasminogen activator (tPA), procalcitonin (PCT), haptoglobin, and alpha-2-macroglobulin (alpha-2-M) (Bio-Rad Laboratories, Hercules, California, USA) (7). Patients were randomised across the series of assays. Sera were diluted as per manufacturers' instructions, except for MMP-2 and−9 which were diluted 1 in 100, and apo-AI, apo-CIII, transthyretin and complement FH which were diluted 1 in 30,000 following optimisation. Assays were performed in single wells with three patients run in duplicate on each plate to estimate intra-assay variability. Quality controls were run on each plate. Plates were read on Bio-Plex 200 instruments at Imperial College London with Bio-Plex Manager v6.1 software (Bio-Rad). Intra-assay variability, calculated as the mean of the coefficients of variance for each analyte individually across all plates, was <12% for all proteins. Results for quality controls fell within expected ranges. If results were below the lower limit of detection, they were assigned a value of zero. If above the upper limit, they were retested at a higher dilution.

### Statistical Analyses

For analyses of individual proteins, all patients with results for that protein were included. Protein concentrations were compared between the TB group and each of the OD and LTBI groups in turn using one-sided Mann-Whitney *U*-tests. The performance of each of the 22 proteins to distinguish TB from each of OD and healthy LTBI in turn by their serum concentration, regardless of HIV status or site, was assessed by the area under the respective ROC curve (ROC AUC). Analyses were performed using GraphPad Prism v7 (GraphPad Software, La Jolla, California, USA).

For the biosignature analyses, as shown in Figure 1, only those patients (i.e., TB and OD) for whom data was gathered for all 22 proteins were included (*n* = 249). This was because a finite number of kits were purchased at the outset, hence if serum from any patient had to be re-tested because a protein concentration was too high, the total number of patients with results for that protein was reduced. Healthy LTBI controls were omitted from these analyses. Patients were classified as TB if the model predicted the probability of TB was >0.5 (*p* > 0.5).

To retest the seven-protein signature from the AE-TBC study, data on the entire AE-TBC cohort were used for discovery (*n* = 701) and on this sample of the ILULU-TB cohort for validation (*n* = 249). The same method was used as for the AE-TBC signature [Generalised Discriminant Analyses (GDA)] using Statistica (Statsoft, Ohio, USA) (7).

For discovery of the optimal new signature, data on the ILULU-TB cohort alone was used. For consistency with the AE-TBC study, patients were randomly allocated to training and test sets at a ratio of 70:30, regardless of HIV status or study site. The same signature discovery methods were also used, namely GDA and Random Forest analyses of log-transformed values. In addition, we also performed variable selection using the Parallel Regularised Regression Model Search method (PReMS) on decile-normalised values using “R” v3.2.2 (R Foundation for Statistical Computing, Vienna, Austria). This is a logistic regression-based method designed to minimise the number of biomarkers selected (10). For each method, the same allocation of patients to training and test sets was used. Assuming the AE-TBC signature had the same accuracy in our data, we had 95% power to show a sensitivity of >90% and specificity of >66.5% with these new signatures.

For a screening test, albeit for community settings, the WHO recommend a minimum sensitivity of 90% (11). No criteria for a rule-in test are specified. After obtaining the best new signature from each method, we therefore re-tested them after adjusting the cut-off for diagnosis to increase each of the sensitivity and specificity in turn to 90%. This was to assess the performance of each signature as either a rule-out or rule-in test for TB. There were no indeterminate test results.

### Ethics Statement

Ethical approval for this study was covered by the approvals for the ILULU-TB study: the Human Research Ethics Committee of the University of Cape Town, South Africa (HREC012/2007), the National Health Sciences Research Committee, Malawi (NHSRC/447), and the Ethics Committee of the London School of Hygiene and Tropical Medicine (5212).

## Results

Demographic and clinical features of individuals selected for this study are shown in Table 1. The range of diagnoses that comprised the OD group is shown in Table 2. Medians and interquartile ranges of proteins in each group are shown in Supplementary Table 1.

**Table 2:** Major clinical diagnoses in the Other Diseases groups of the sample of the ILULU-TB cohort that was selected for this study.

	**HIV uninfected**		**HIV infected**		**Total** **(% of OD group)**
	**Karonga**	**Cape Town**	**Karonga**	**Cape Town**	
Pneumonia/ Bronchitis/PCP	12 (33%)	6 (16%)	15 (47%)	15 (38%)	48 (33%)
Malignancy or neoplasia other than KS^*^	3 (8%)	13 (35%)	1 (3%)	2 (5%)	19 (13%)
Genitourinary	6 (17%)	4 (11%)	2 (6%)	1 (3%)	13 (9%)
Meningitis (bacterial/viral/ unspecified)	4 (11%)	0 (0%)	4 (13%)	2 (5%)	10 (7%)
Gastroenteritis/ Hepatitis	0 (0%)	2 (5%)	0 (0%)	6 (15%)	8 (6%)
Kaposi's Sarcoma	0 (0%)	0 (0%)	1 (3%)	6 (15%)	7 (5%)
Pyelonephritis	0 (0%)	7 (19%)	0 (0%)	0 (0%)	7 (5%)
Cryptococcal meningitis	0 (0%)	0 (0%)	2 (6%)	3 (8%)	5 (3%)
Pleural effusion/empyema (non-TB)	0 (0%)	1 (3%)	0 (0%)	4 (10%)	5 (3%)
Bacteraemia (source not identified)	1 (3%)	0 (0%)	4 (13%)	0 (0%)	5 (3%)
Other^**^	5 (14%)	0 (0%)	0 (0%)	0 (0%)	5 (3%)
Hepatobiliary disease	0 (0%)	4 (11%)	0 (0%)	0 (0%)	4 (3%)
Peritonitis	3 (8%)	0 (0%)	1 (3%)	0 (0%)	4 (3%)
Malaria	1 (3%)	0 (0%)	1 (3%)	0 (0%)	2 (1%)
IBD	0 (0%)	0 (0%)	0 (0%)	1 (3%)	1 (1%)
Pyomyositis	1 (3%)	0 (0%)	0 (0%)	0 (0%)	1 (1%)
Persistent generalised lymphadenopathy	0 (0%)	0 (0%)	1 (3%)	0 (0%)	1 (1%)
**TOTAL**	**36**	**37**	**32**	**40**	145

*Data are stratified by HIV status and site*.

* *Lung cancer (n = 7), lymphoma (n = 3), dermatological tumour (n = 2), unspecified (n = 2), mesothelioma (n = 1), hepatocellular carcinoma (n = 1), metastatic adenocarcinoma (n = 1), benign salivary gland tumour (n = 1)*.

** *Epilepsy (n = 3), headache (n = 1), pain unspecified (n = 1). One patient had no diagnosis listed, hence data is shown for 145 patients*.

### Performance of Biomarkers Individually

**Figure 2:**
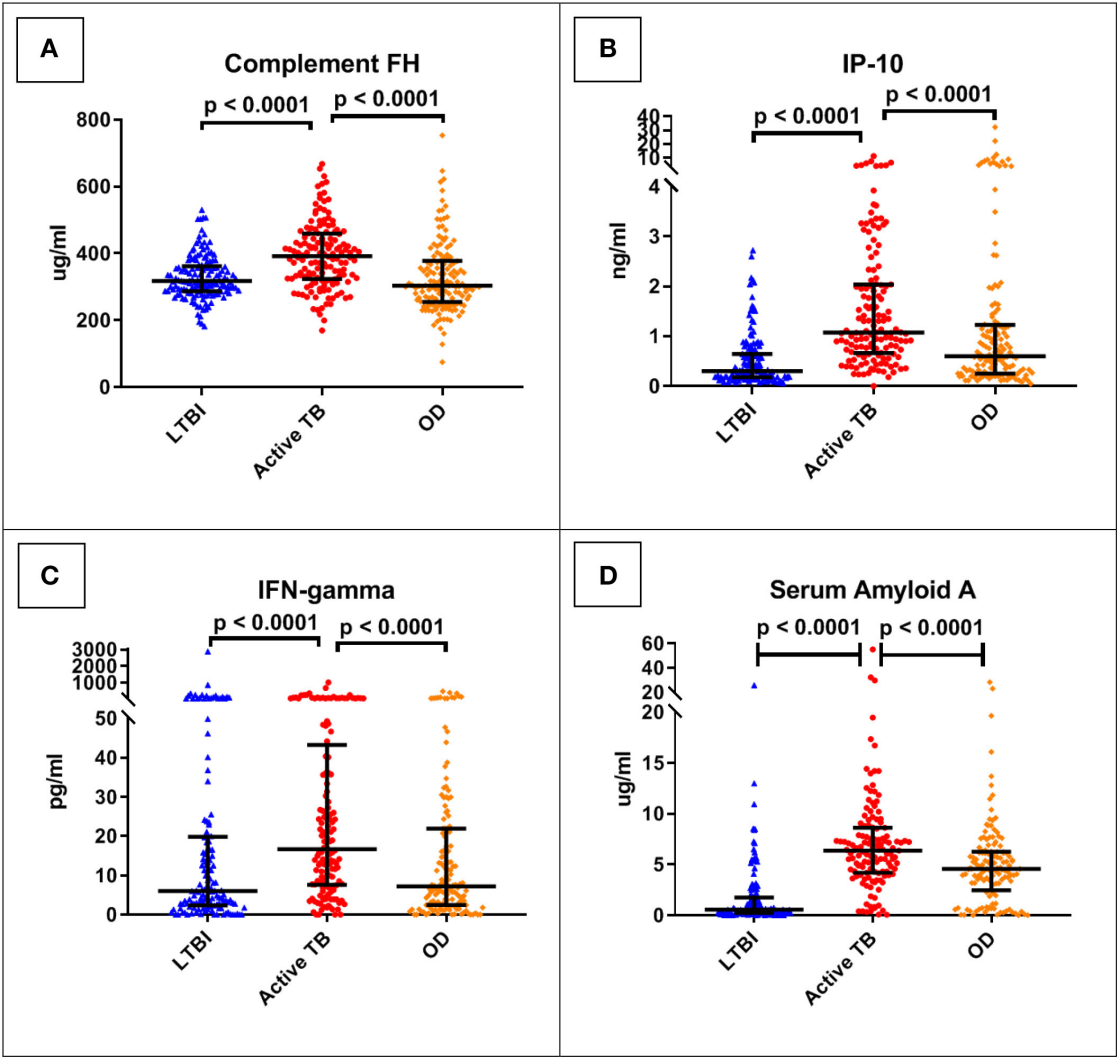
Serum concentrations of the top four protein biomarkers (panels **A–D**) by clinical group. Scatter-dot plots show results for each patient in the ILULU-TB cohort, regardless of HIV status or site. *P*-values are 1-sided and derived from Mann-Whitney tests. Error bars represent medians and interquartile ranges. IP-10, IFN-gamma-inducible protein 10; IFN-gamma, interferon-gamma.

The best performing protein was complement factor H (FH). As shown in Table 3, this had a ROC AUC of 70% (95% confidence interval (CI): 64–76%). This performance was preserved across the sites (70% in Cape Town, 71% in Karonga) and HIV status (71% in HIV uninfected, 69% in HIV infected). ROC curves for these subdivisions are shown in Supplementary Figure 1. In addition, as shown in Figure 2, in comparison with the healthy LTBI control group, concentrations were higher in the TB group but trended toward being lower in the OD group (*p* = 0.072). This contrasted with the other 21 proteins, in which concentrations in the TB and OD groups differed from those in the LTBI group in the same direction.

**Table 3:** Diagnostic accuracy of protein biomarkers individually.

**Protein**	**Number tested from ILULU-TB cohort**	**ROC AUC in ILULU-TB study (%)**	**ROC AUC in AE-TBC study (%)**
Complement FH	292	70 (64–76)	58 (53–62)
IP-10	282	66 (60–73)	82 (79–86)
IFN-gamma	284	66 (60–72)	80 (76–84)
SAA	263	65 (58–71)	83 (80–86)
VEGF	278	64 (57–71)	70 (65–74)
Haptoglobin	263	64 (58–71)	62 (57–66)
SAP	267	64 (57–71)	58 (53–63)
Transthyretin	292	61 (55–68)	78 (74–82)
Apo-CIII	292	58 (51–64)	65 (61–70)
Ferritin	263	57 (50–64)	78 (75–82)
tPA	263	57 (50–64)	72 (68–76)
Alpha-2-M	267	57 (50–64)	54 (49–58)
Fibrinogen	263	56 (49–63)	73 (69–77)
TGF-alpha	284	55 (49–62)	73 (69–77)
TNF-alpha	284	53 (46–59)	69 (65–74)
MMP-9	292	53 (47–60)	59 (53–64)
Apo-AI	292	52 (45–59)	69 (65–73)
Procalcitonin	263	52 (45–59)	68 (63–72)
IFN-alpha-2	284	52 (45–58)	67 (62–71)
MMP-2	292	52 (45–58)	54 (49–59)
CRP	267	51 (43–58)	84 (81–87)
IL-1RA	283	51 (44–58)	63 (58–68)

*Areas under the receiver operating characteristic curve (ROC AUCs) for the performance of each protein in distinguishing TB (n = 146) from OD (n = 146) are shown, regardless of HIV status or site. Proteins are listed in descending order of performance in the ILULU-TB cohort with the numbers of patients for which results were obtained for that protein. ROC AUCs are shown as percentages. Results from the AE-TBC study are shown to the right for comparison (6). Bracketed values indicate 95% confidence intervals*.

The concentrations of the top four individual biomarkers in each group are shown in Figure 2, and a display of all individual ROC AUCs is shown in Table 3. In comparison with the AE-TBC study, four proteins performed better in the ILULU-TB cohort (complement FH, SAP, haptoglobin, and alpha-2-M). The remaining 18 showed inferior performance, and the protein with the largest drop in performance was CRP, which was the best performing biomarker in the AE-TBC study and part of the seven-protein signature. Individual ROC AUCs in order of their difference compared to the AE-TBC study are shown in Supplementary Figure 2.

**Figure 3:**
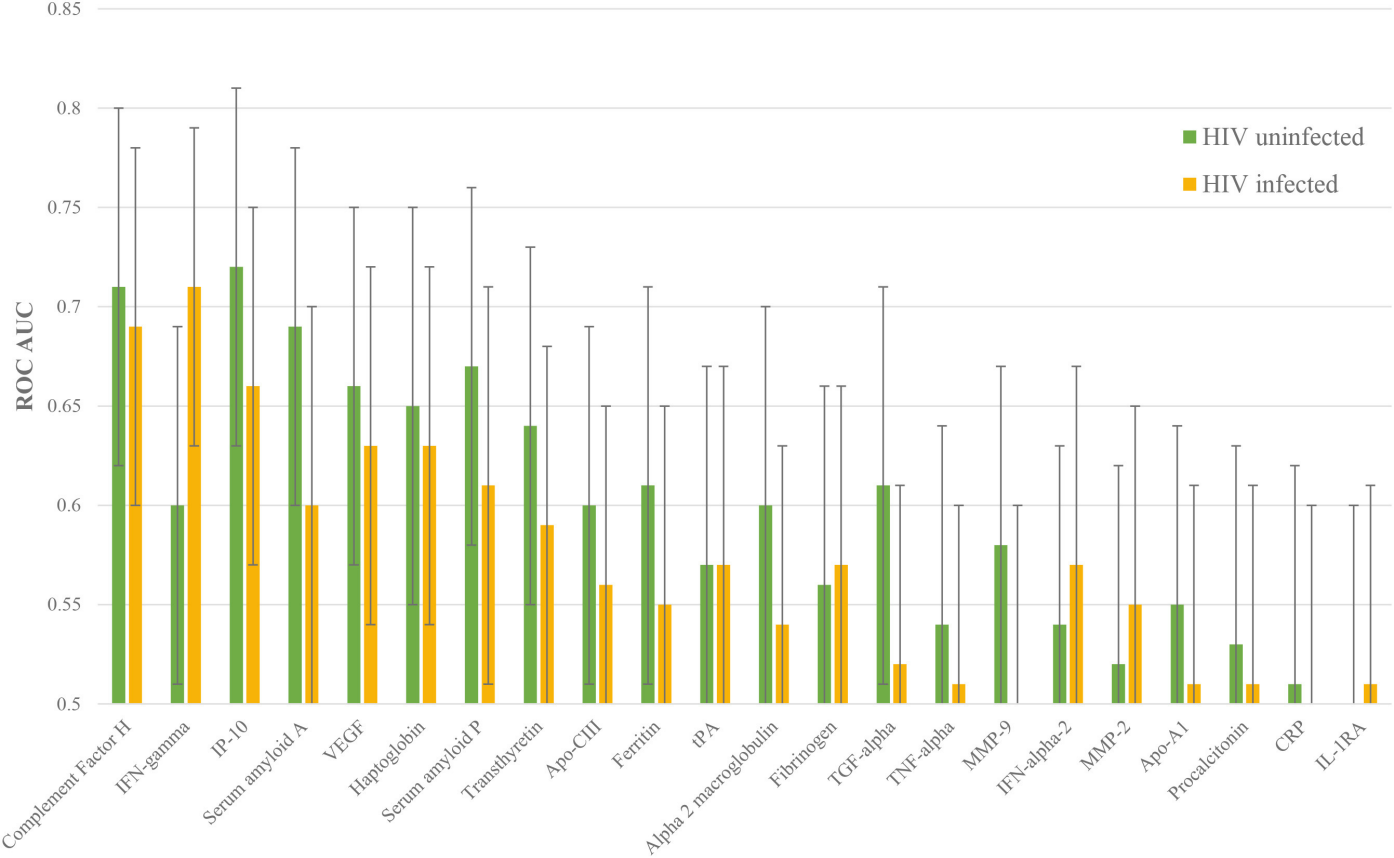
Individual ROC AUCs for each of HIV uninfected and infected halves of this sample of the ILULU-TB cohort. Green bars: distinction of TB, HIV– from OD, HIV–; yellow bars: TB, HIV+ from OD, HIV+.

The performance of each protein was then stratified by HIV status. 16 proteins performed better in HIV uninfected patients: complement FH, IP-10, SAA, VEGF, haptoglobin, SAP, transthyretin, apo-CIII, ferritin, alpha-2-M, TGF-alpha, TNF-alpha, MMP-9, apo-AI, PCT, and CRP. Five proteins performed better in HIV co-infected patients: IFN-gamma, fibrinogen, IFN-alpha-2, MMP-2, and IL-1RA. Confidence intervals overlapped for every protein, however (Figure 3).

Finally, while the main aim was to assess performance of proteins to distinguish TB from OD, we also examined their performance to distinguish TB from LTBI. The protein with the highest ROC AUC for this purpose was CRP (92%, Supplementary Table 2).

### Performance of the AE-TBC Signature in the ILULU-TB Cohort

For the biosignature analyses, 122 TB patients and 127 OD patients for whom results were available for all 22 proteins were included. There was an equal distribution of patients across the clinical groups, sites, and HIV status (Figure 1).

The performance of the seven-protein signature from the AE-TBC study in the ILULU-TB cohort is shown in Table 4. With the cut-off for defining a positive test at the default setting (*p* > 0.5), the sensitivity was greater than in the AE-TBC study [98% (95% CI: 94–100%)], but specificity was markedly reduced [12% (7–19%)]. On comparison of biomarker concentrations between studies, there were significant differences in some proteins, especially apo-AI (Supplementary Figure 3). To understand this, we compared concentrations of apo-AI in our healthy LTBI controls with published normal concentrations. Concentrations in our LTBI group were 4-fold lower than those published (medians 324 vs. 1,180 ug/ml) (12). Concentrations of apo-CIII, however, which was part of the same multiplexed panel, matched those published (medians 114 vs. 114 ug/ml) (13). In addition, concentrations of apo-AI in the AE-TBC TB cohort were higher than published normal concentrations (2,000 vs. 1,180 ug/ml), even though apo-AI concentrations decrease in TB (7).

**Table 4:** Performance of the seven-protein signature from the AE-TBC study in the ILULU-TB cohort.

	**AE-TBC cohort (discovery set)**	**ILULU-TB cohort (validation set)**
ROC AUC	91 (89–93)	
Sensitivity	85 (80–90)	98 (94–100)
Specificity	85 (81–88)	12 (7–19)
PPV	71 (65–76)	52 (45–58)
NPV	93 (90–95)	88 (64–99)

*For this analysis, the entire AE-TBC cohort was used as the discovery set (n = 701), and the whole sample of the ILULU-TB cohort as the validation set (n = 249). Results are shown with the threshold for defining a case of TB at the default setting (p > 0.5). All results are given as percentages. PPV, positive predictive value. NPV, negative predictive value. Bracketed values represent 95% confidence intervals*.

### Performance of New Signatures Derived in the ILULU-TB Cohort

The numbers of patients in each subgroup of the ILULU-TB cohort that were randomised to each of the train and test sets are shown in Table 5. The same patients were used for this set of analyses as for the re-test of the AE-TBC signature (*n* = 249: 122 TB and 127 OD). The results of the best new signatures from each of the GDA, Random Forests and PReMS methods are shown in Tables 6–8. Results are shown with the cut-off at the default setting, and after increasing each of sensitivity and specificity in turn to 90%. Positive and negative predictive values (PPV and NPV) are also shown in each case, based on the equal sizes of the TB and OD groups in this study.

**Table 5:** Numbers of patients in the ILULU-TB cohort allocated to train and test sets, for novel biosignature discovery.

**Site**	**Clinical group**	**Train (70%)**	**Test (30%)**	**TOTAL**
Karonga	TB, HIV–	29	3	32
	TB, HIV+	14	17	31
	OD, HIV–	11	20	31
	OD, HIV+	24	4	28
Cape Town	TB, HIV–	21	9	30
	TB, HIV+	21	8	29
	OD, HIV–	27	4	31
	OD, HIV+	27	10	37
**TOTAL**		**174**	**75**	249

*Numbers in each of the clinical subgroups and at each study site are shown. Patients were allocated to train or test set at random. Only those patients with results for all 22 proteins were included*.

**Table 6:** Performance of a new five-protein signature derived from Generalised Discriminant Analyses (GDA).

	**Training set**	**Test set**
ROC AUC	84 (78–90)	
Sensitivity	82 (73–90)	81 (65–92)
Specificity	74 (64–83)	63 (46–78)
PPV	75 (65–84)	68 (52–81)
NPV	81 (71–89)	77 (59–90)
**After adjustment of cut-off for increased sensitivity**		
Sensitivity	89 (80–94)	79 (63–90)
Specificity	48 (37–59)	41 (25–58)
PPV	64 (55–73)	58 (43–71)
NPV	80 (67–90)	65 (43–84)
**After adjustment of cut-off for increased specificity**		
Sensitivity	61 (50–71)	58 (41–74)
Specificity	91 (82–96)	89 (75–97)
PPV	87 (76–94)	85 (65–96)
NPV	69 (59–77)	67 (52–80)

*This analysis was performed using data on the ILULU-TB cohort only (n = 249). Patients were randomly assigned to training and test sets at a ratio of 70:30. The signature comprised Complement FH, IP-10, CRP, SAA, and Transthyretin. Results are shown both before and after adjusting the probability threshold in the training set for diagnosing TB to increase each of sensitivity and specificity in turn to 90%*.

**Table 7:** Performance of a new 22-protein signature derived from Random Forests analyses.

	**Training set**	**Test set**
Sensitivity	72 (61–81)	73 (56–86)
Specificity	80 (70–88)	71 (54–85)
PPV	77 (66–86)	71 (54–85)
NPV	75 (65–83)	73 (56–86)
**After adjustment of cut-off for increased sensitivity**		
Sensitivity	82 (73–90)	92 (78–98)
Specificity	64 (53–74)	58 (41–74)
PPV	69 (59–77)	68 (53–80)
NPV	79 (68–88)	88 (69–97)
**After adjustment of cut-off for increased specificity**		
Sensitivity	65 (54–75)	43 (27–61)
Specificity	89 (80–94)	95 (82–99)
PPV	85 (74–92)	89 (65–99)
NPV	72 (63–81)	63 (49–76)

*This analysis was performed using data on the ILULU-TB cohort only (n = 249). The same patients with the same allocations to training and test sets were used as for the GDA analyses (Table 4)*.

**Table 8:** Performance of a nine-protein signature derived from Parallel Regularised Regression Model Search.

	**Training set**	**Test set**
ROC AUC	90 (86–95)	84 (73–94)
Sensitivity	83 (75–90)	86 (73–95)
Specificity	82 (73–89)	74 (58–86)
PPV	84 (75–91)	85 (70–94)
NPV	82 (73–89)	76 (62–87)
**After adjustment of cut-off for increased sensitivity**		
Sensitivity	90 (83–95)	92 (80–98)
Specificity	71 (61–80)	71 (56–84)
PPV	75 (66–82)	75 (62–86)
NPV	89 (80–95)	90 (76–97)
**After adjustment of cut-off for increased specificity**		
Sensitivity	73 (63–82)	75 (60–87)
Specificity	90 (82–95)	81 (67–91)
PPV	87 (78–94)	80 (65–91)
NPV	78 (69–85)	77 (63–88)

*This analysis was performed using data on the ILULU-TB cohort only (n = 249). The same patients and allocations were used as for the GDA and Random Forests analyses (Tables 4, 5). This signature comprised fibrinogen, alpha-2-M, CRP, MMP-9, transthyretin, complement FH, IFN-gamma, IP-10, and TNF-alpha*.

The GDA method yielded a five-protein signature comprising complement factor H, IP-10, CRP, SAA, and transthyretin. The ROC AUC in the training set was 84% (Table 6). Sensitivities and specificities in the test set were 81% and 63% initially, 79% and 41% after increasing sensitivity, and 58% and 89% after increasing specificity.

The results of the Random Forests analyses, using all 22 proteins, are shown in Table 7. Sensitivities and specificities in the test set were 73% and 71% initially, 92% and 58% after increasing sensitivity, and 95% and 43% after increasing specificity.

The PReMS method yielded a nine-protein signature comprising fibrinogen, alpha-2-M, CRP, MMP-9, transthyretin, complement FH, IFN-gamma, IP-10, and TNF-alpha. As shown in Table 8, this had a ROC AUC of 90% in the training set and 84% in the test set. Sensitivities and specificities in the test set were 86% and 74% initially, 92% and 71% after increasing sensitivity, and 75% and 81% after increasing specificity. At the cut-off for increased sensitivity, PPV and NPV in the test set were 75% and 90%, respectively. The performance in each of the HIV uninfected and co-infected halves of the test set in terms of ROC AUC was 84% for HIV uninfected patients (95% CI: 72–97%) and 86% for co-infected patients (95% CI: 71–100%), regardless of site. The ROC AUC at each of the two sites was 94% at Cape Town (95% CI: 86–100%) and 78% at Karonga (95% CI: 63–93%), regardless of HIV status. The difference between the ROC AUCs at the two sites was not significant by DeLong's test, however (*p* = 0.069) (14).

## Discussion

In the field of host serum proteomics-based TB diagnostics, this study stands out for several reasons. Firstly, it was conducted in Africa, where the burden of TB is highest, and included equal numbers of patients with and without HIV. This is important because the host response to TB may vary by ethnicity (15, 16), and is also distinct in the setting of HIV co-infection. Differences in concentrations of serum proteins between TB patients with and without HIV co-infection have not been extensively studied, although concentrations of neopterin and beta-2-microglobulin have both been found to be significantly higher in TB patients with HIV than without (17). This may reflect a state of “immune activation” in HIV-associated TB, which is well-recognised (18–20). Fundamentally, however, the pathogenesis of TB in HIV co-infection differs significantly, with impaired granuloma formation, less pulmonary cavitation and more dissemination (21–23). With the prevalence of HIV amongst patients presenting with active TB as high as 50% in some areas of Africa, and the TB case fatality rate in HIV co-infection being approximately twice that of HIV uninfected individuals (1), it is essential that any biosignature for use in such settings be derived from a representative population. The range of other diseases to be distinguished from TB is also strongly associated with both geographical location and HIV prevalence. Previous studies have derived promising serum protein signatures using techniques including mass spectrometry, but these were either not set in Africa or did not include or amalgamate sufficient numbers of HIV co-infected patients in both TB and OD (or control) groups (24–29). The two sites in Cape Town and Karonga were also selected in this study to represent the spread of epidemiological settings in Africa. Cape Town was selected to represent urban sites, and also had a low prevalence of malaria. Karonga was selected as a rural site, and had a high prevalence of malaria and other parasitic infections. The second major strength of this study was that patients were prospectively recruited from a point of differential diagnosis. An early study by Agranoff et al. included African sera, but the OD group comprised a selection of diseases whose clinical features “can overlap with” those of TB (26). This is less rigorous, since a population which is more homogenous clinically (such as ours) is likely to be more homogenous in their serum proteomes, and therefore a more challenging one from which to derive markers of host response that are TB-specific. Thirdly, our signatures were tested using immunocapture. Whilst not arising from an untargeted proteomic approach, this ensured that any such signatures were more easily translatable to lateral flow immunoassay. To our knowledge, none of the relevant mass spectrometry-based studies published in the literature performed full technical validation by immunocapture (24–28). Fourthly, the patients recruited to this study were largely hospital attendees, which is also a population currently under-represented in the literature. Two studies recruited hospital patients from sites including in Africa, but either did not include HIV co-infected patients in the discovery cohort, or had a low number of HIV infected patients in the OD group (25, 26). Several recent studies have employed immunocapture to discover new signatures including in patients from Africa, but recruitment was limited to primary care settings (29–33). Patients presenting to hospitals are likely to be more unwell than those presenting to primary care settings, and therefore to have a greater degree of disturbance to their serum proteome. The TB patients in Cape Town were recruited from a clinic, however all were culture positive as per the study design, and therefore likely to have had more advanced disease than cohorts that included clinical diagnoses. Severity of TB is known to affect the concentrations of serum proteins, including CRP, procalcitonin, and serum amyloid A, hence the importance of evaluating biomarker performance at this different level of the healthcare system (34, 35). Other strengths of the study design were that diagnoses were confirmed in all patients, and that healthy controls with LTBI infection were included for reference.

The design of this study was also well-suited to re-testing the biomarkers from the AE-TBC study. The countries in which recruitment took place were a subset of the countries in the AE-TBC study (Malawi and South Africa); the assays were performed using the same Luminex kits and analyser; and the same statistical methods were applied to the data, by the same statistician (7). To complement the signature discovery process, an additional method (PReMS) was also used.

Limitations included the fact that even though the recruitment process was open to extrapulmonary TB (EPTB) cases, no cases of culture positive TB without pulmonary involvement were included. In addition, none of the OD cases were documented as having non-tuberculous mycobacterial disease (NTM), which may more closely resemble TB in terms of host response (36). Secondly, this study was limited to the 22 proteins that had been selected by the AE-TBC based on previous performance in primary care settings. This was a strength in that the biomarkers had been through prior selection to diagnose the disease of interest (TB), but a weakness since they had not all previously been selected from presentations to secondary care. A third weakness was that, in terms of the comparison of biomarker performance between the ILULU-TB and AE-TBC studies, the study designs were different: ILULU-TB was case-control, with group sizes held equal, whereas AE-TBC was a cohort study. The group sizes in the latter therefore reflected local epidemiology, including with regards to HIV prevalence. Another difference was that our OD group comprised both pulmonary and non-pulmonary diseases, whereas AE-TBC focussed on lung diseases only. A final limitation was that our study did not include an external cohort in which to validate any new signatures.

Overall, the performance of the proteins individually was less good in the ILULU-TB cohort, except for complement FH, SAP, haptoglobin, and alpha-2-M. The results for complement FH were particularly promising in that diagnostic performance was sustained across site and HIV status. In addition, the fact that concentrations of complement FH in the TB and OD groups moved in a different direction from each other relative to the healthy controls implies that rising concentrations of complement FH may be TB-specific. Complement FH did distinguish TB from OD (or “no-PTB”) in the AE-TBC study, with higher concentrations in the TB group, but this difference was more pronounced in the ILULU-TB cohort. A possible reason for that might be that transcription of complement FH *in vitro* is driven by IFN-gamma (37, 38), which in turn has a central role in the host response in TB (39, 40). In keeping with this, IFN-gamma serum concentrations were higher in our TB group than OD group (Figure 2C). As discussed above, the TB cases in the ILULU-TB study were likely more advanced than those in the AE-TBC study, which may have driven serum FH concentrations up higher. An additional possibility is that FH concentrations were lower in our OD group, again due to more severe illnesses. Complement FH concentrations in serum/plasma have not been extensively studied in other infections, but are known to decrease in inflammatory conditions such as lupus nephritis and myaesthenia gravis as a result of excessive complement consumption (41, 42). Enhanced complement activation and consumption has also been shown to occur in HIV-infected patients with sepsis (43), and this may have been relevant for a proportion of our OD cohort. By contrast, the particularly poor performance of CRP in this study is interesting, since this was the best-performing biomarker individually in the AE-TBC study, with concentrations being higher in the TB group. Whilst concentrations trended toward being higher in the TB group in the ILULU-TB study, this difference was not statistically significant, and CRP did not function as a standalone biomarker. This was likely reflective, again, of the more severely ill state of the OD patients in the ILULU-TB cohort, rather than any reduction of levels in our TB cohort. This is supported by CRP being the top individual biomarker to distinguish TB from LTBI in our cohort, and also by previous observations that CRP performs significantly less well in hospital than in community settings (44, 45).

The application of the 7-protein signature from the AE-TBC study directly to the data from the ILULU-TB study was hampered by the fact that concentrations of some of the proteins differed significantly between the two studies. Data accuracy and precision within each of the two studies was good, which suggests that the commonly observed phenomenon of lot-lot variation between multiplexed kits was the main contributor (46). It is possible that the marked decrease in concentrations of apo-AI in our study represent over-correction of calibration by the manufacturer of previously high concentrations, such as were reported in the AE-TBC study.

The newly derived 5-protein GDA signature had a moderately high ROC AUC in the training set of 84% (78–90%). In the test set, however, sensitivity and specificity were less promising. The five proteins were a subset of the seven that comprised the AE-TBC signature, however, which validates them as being among the best biomarkers for TB diagnosis. The Random Forests method produced performances in the test set that were slightly greater, but this was with all 22 proteins included in the model, which is less feasible for translation to a POC test.

The best performing test emerged from the PReMS method in the form of a nine-protein signature comprising fibrinogen, alpha-2-M, CRP, MMP-9, transthyretin, complement factor H, IFN-gamma, IP-10, and TNF-alpha. The highest combined results came from optimising the sensitivity, which yielded 92% sensitivity and 71% specificity in the test set. This was comparable with the performance of the seven-protein signature in the AE-TBC study. It also exceeded the WHO minimum requirements for a “triage test” for TB, which is notable, even though that particular target was designed with community settings in mind (11). The potential benefit of a screening test in hospital settings is clear, since it would decrease the number of sputum-based investigations that would be needed, including by GeneXpert, as well as unnecessary courses of TB treatment. The performance of the signature was unaffected by HIV status, which is promising for use in African settings, and also contrasts with the performance of sputum smear microscopy, which is significantly less sensitive in HIV co-infected patients (47).

This study focussed on culture positive TB, in order to derive a signature based on confirmed cases. Future validation studies, however, should include culture negative pulmonary TB cases, as well as EPTB, and OD groups including NTM disease. In addition, translation to POC will depend on the availability of LFA platforms for measuring multiple proteins. LFAs have been shown to be feasible for use in sub-Saharan African settings and accurate across four orders of magnitude, without the need for a cold chain for distribution or storage (6). Multiplexing technology is also emerging for LFAs, with multiple proteins either being detected in series, along one strip (48, 49), or in parallel, with multiple strips contained within one handheld device (50).

In summary, we retested the performance of 22 host serum protein biomarkers of TB that had originally been selected from primary care studies in Africa in a large sample from a well-characterised cohort recruited largely from hospitals. The top-performing single biomarker was complement factor H, which is a novel marker of TB in this setting. A nine-protein biosignature was discovered which showed promise for use as a POC screening test in hospital settings, and performed equally well in individuals co-infected with HIV. Translation to this will depend on validation in independent cohorts and on development of accurate POC platforms.

## Data Availability Statement

The raw data supporting the conclusions of this article will be made available by the authors, without undue reservation.

## Ethics Statement

The studies involving human participants were reviewed and ethical approval for this study was covered by the approvals for the ILULU-TB study: the Human Research Ethics Committee of the University of Cape Town, South Africa (HREC012/2007), the National Health Sciences Research Committee, Malawi (NHSRC/447), and the Ethics Committee of the London School of Hygiene and Tropical Medicine (5212). The patients/participants provided their written informed consent to participate in this study.

## Author Contributions

HD, ML, RW, and MH: conceived and designed the experiments. TM: performed the experiments. TM, CH, and MK: analysed the data. TM, CH, NC, MK, NF, GW, ML, RW, and MH: provided input into data interpretation. TM, CH, NC, RW, and MH: contributed to writing the first version of the manuscript. NC, TO, KW, HD, AC, NF, RW, and MH: contributed to revisions of the manuscript. TO, RG, LS, LB, ML, and RW: enrolled patients used in this study. TO, RG, KW, LS, and AC: data collection on patients used in this study. All authors contributed to the article and approved the submitted version.

## Conflict of Interest

The authors declare that the research was conducted in the absence of any commercial or financial relationships that could be construed as a potential conflict of interest.
