# Supplementary material for: Evaluation of Host Serum Protein Biomarkers of Tuberculosis in sub-Saharan Africa
Source: Front Immunol. 2021 Feb 25;12:639174. doi: 10.3389/fimmu.2021.639174 (PMC7947659; doi:10.3389/fimmu.2021.639174)
Supplement: Supplementary file 1 [file Data_Sheet_1.docx]

**Supplementary Information**

**Supplementary Table 1. Serum concentrations of each protein in each of the three main clinical groups.** Data on the sample of the ILULU-TB that was taken for this study are shown regardless of HIV status and site. TB: active tuberculosis; OD: other diseases; LTBI: latent TB. IQR: interquartile range. Units of protein concentration: mg/ml: Alpha-2-M and Haptoglobin; ug/ml: fibrinogen, serum amyloid A, CRP, serum amyloid P, MMP-2, MMP-9, apo-AI, apo-CIII, transthyretin, and complement FH; ng/ml: procalcitonin, ferritin, tPA, and IP-10; and pg/ml: TGF-alpha, IFN-alpha-2, IFN-gamma, IL-1RA, TNF-alpha, and VEGF.

|  | **TB** | | **OD** | | **LTBI** | |
| --- | --- | --- | --- | --- | --- | --- |
|  | **Median** | **IQR** | **Median** | **IQR** | **Median** | **IQR** |
| **Procalcitonin** | 1·90 | (1·20 - 2·64) | 1·79 | (1·20 - 2·87) | 1·20 | (0·73 - 1·79) |
| **Ferritin** | 74·45 | (30·50 - 139·55) | 54·32 | (20·93 - 135·91) | 11·78 | (5·59 - 20·18) |
| **tPA** | 2·72 | (1·86 - 3·74) | 2·22 | (1·65 - 3·30) | 1·29 | (0·92 - 2·03) |
| **Fibrinogen** | 2·71 | (2·01 - 3·46) | 2·34 | (1·51 - 3·52) | 1·65 | (0·93 - 2·32) |
| **SAA** | 6·36 | (4·23 - 8·59) | 4·58 | (2·83 - 6·25) | 0·55 | (0·23 - 1·73) |
| **Alpha-2-M** | 1·44 | (1·22 - 1·77) | 1·36 | (1·09 - 1·62) | 1·49 | (1·19 - 1·76) |
| **CRP** | 63·35 | (29·20 - 100·12) | 45·51 | (9·55 - 163·47) | 1·01 | (0·54 - 3·28) |
| **Haptoglobin** | 4·83 | (0·35 - 12·75) | 1·38 | (0·28 - 5·70) | 1·33 | (0·20 - 3·43) |
| **SAP** | 42·28 | (34·19 - 52·28) | 35·60 | (24·82 - 47·74) | 31·22 | (25·17 - 38·05) |
| **MMP-2** | 77·70 | (63·56 - 100·63) | 79·71 | (59·99 - 110·24) | 110·66 | (85·30 - 134·00) |
| **MMP-9** | 335·97 | (195·54 - 516·25) | 276·52 | (149·94 - 571·16) | 210·23 | (106·45 - 317·56) |
| **Apo-AI** | 230·81 | (171·61 - 291·63) | 241·75 | (164·52 - 335·23) | 324·44 | (253·91 - 418·56) |
| **Apo-CIII** | 85·49 | (66·71 - 122·84) | 105·62 | (68·025 - 147·09) | 113·88 | (87·45 - 145·40) |
| **Transthyretin** | 69·64 | (48·80 - 104·55) | 89·65 | (59·68 - 158·53) | 189·83 | (129·33 - 260·92) |
| **Complement FH** | 391·88 | (324·43 - 455·99) | 303·87 | (257·64 - 376·53) | 317·51 | (287·54 - 359·88) |
| **TGF-alpha** | 10·37 | (6·01 - 19·37) | 8·32 | (2·94 - 18·09) | 5·35 | (2·56 - 11·17) |
| **IFN-alpha-2** | 0·00 | (0·00 - 33·50) | 0·00 | (0·00 - 35·88) | 0·00 | (0·00 - 14·51) |
| **IFN-gamma** | 16·31 | (7·70 - 40·85) | 6·79 | (2·35 - 20·65) | 5·94 | (2·33 - 18·92) |
| **IL-1RA** | 16·71 | (2·59 - 49·02) | 14·56 | (0·00 - 75·25) | 1·10 | (0·00 - 17·85) |
| **IP-10** | 1·08 | (0·66 - 2·04) | 0·60 | (0·25 - 1·20) | 0·30 | (0·18 - 0·65) |
| **TNF-alpha** | 25·04 | (15·90 - 39·73) | 22·04 | (13·25 - 31·95) | 16·02 | (10·75 - 24·02) |
| **VEGF** | 291·97 | (135·80 - 674·27) | 157·41 | (61·09 - 307·75) | 134·09 | (70·81 - 265·27) |

**Supplementary Figure 1.** **ROC curves for the accuracy of complement factor H in this sample of the ILULU-TB cohort after stratification by each of HIV status and site.** All patients with results for complement FH were included: 146 with TB and 146 with OD. HIV-: HIV uninfected; HIV+: HIV infected

| 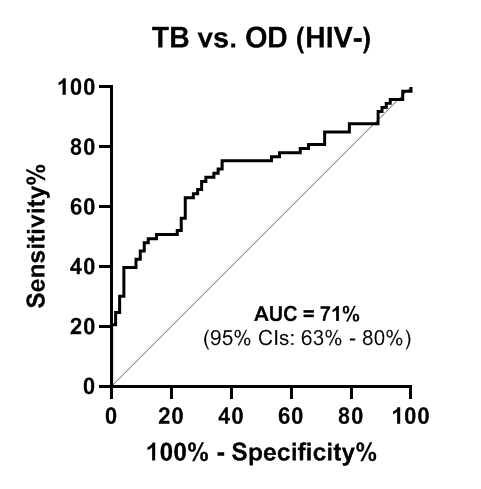 | 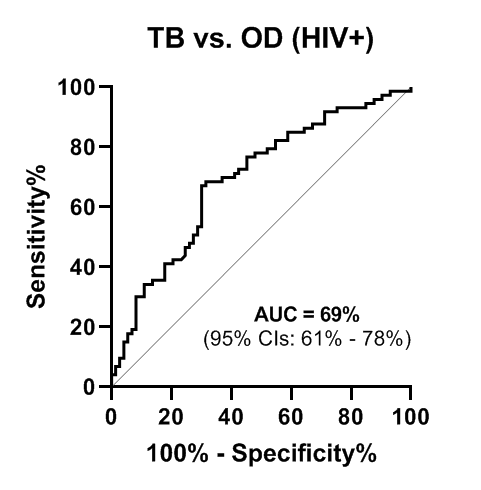 |
| --- | --- |
| 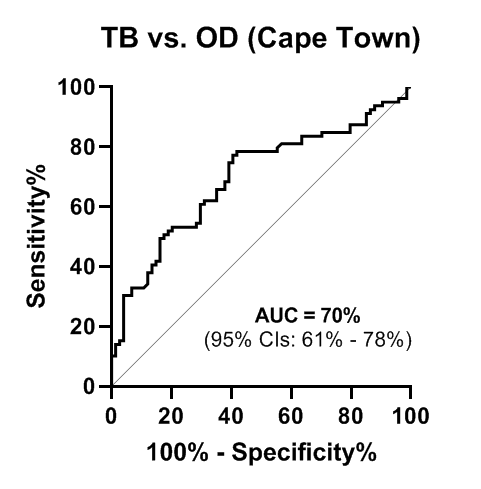 | 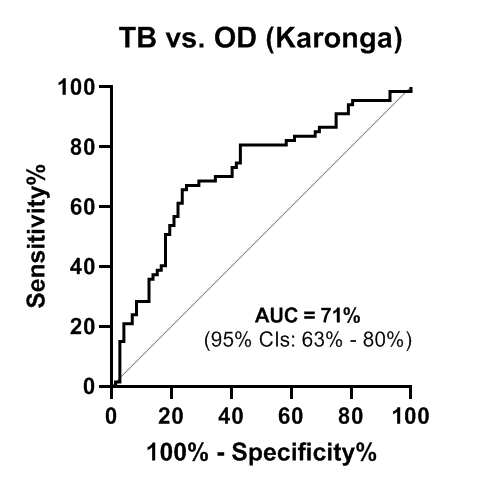 |

**Supplementary Figure 2. ROC AUCs of each protein to distinguish TB from OD in order of the size of the difference in performance between the two studies.** Orange bars: performance in this sample of the ILULU-TB cohort; blue bars: performance in AE-TBC cohort. Error bars represent 95% confidence intervals. Asterisks indicate proteins that belonged to the AE-TBC seven protein signature.

**Supplementary Table 2. ROC AUCs for each of the proteins to distinguish active TB from LTBI**. Results are shown on this sample of the ILULU-TB cohort regardless of HIV status or site. Proteins are listed in descending order of diagnostic performance. The number of patients with both TB and LTBI that had results for each protein are shown in the right-hand column.

|  | **ROC AUC (%)**  **(95% CI)** | **Number tested** |
| --- | --- | --- |
| **CRP** | 92 (88-95) | 267 |
| **Ferritin** | 88 (84-92) | 265 |
| **SAA** | 87 (83-92) | 265 |
| **Transthyretin** | 86 (82-90) | 292 |
| **IP-10** | 83 (78-87) | 279 |
| **tPA** | 79 (74-85) | 265 |
| **SAP** | 75 (69-81) | 267 |
| **Fibrinogen** | 75 (69-80) | 264 |
| **Apo-AI** | 73 (68-79) | 292 |
| **MMP-2** | 73 (67-79) | 292 |
| **Complement FH** | 70 (64-76) | 292 |
| **Haptoglobin** | 69 (63-76) | 262 |
| **PCT** | 69 (63-75) | 265 |
| **TGF-alpha** | 69 (63-75) | 281 |
| **VEGF** | 68 (61-74) | 277 |
| **MMP-9** | 68 (62-74) | 292 |
| **TNF-alpha** | 67 (60-73) | 281 |
| **IFN-gamma** | 66 (59-72) | 281 |
| **IL-1RA** | 66 (59-72) | 281 |
| **Apo-CIII** | 65 (58-71) | 292 |
| **IFN-alpha-2** | 56 (49-63) | 282 |
| **Alpha-2-M** | 51 (44-58) | 267 |

**Supplementary Figure 3. Comparison of serum concentrations of each protein in the TB groups between the two studies**. AE-TBC: African European TB Consortium. ILULU-TB: European Union-funded Imperial College-led TB biomarker study. Boxes represent medians with interquartile ranges. Asterisks indicate proteins that belong to the AE-TBC seven-protein signature.

| 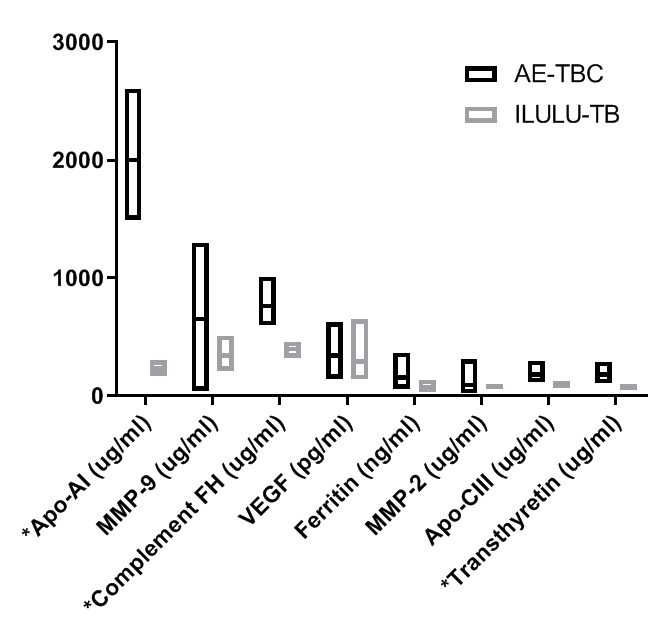 | 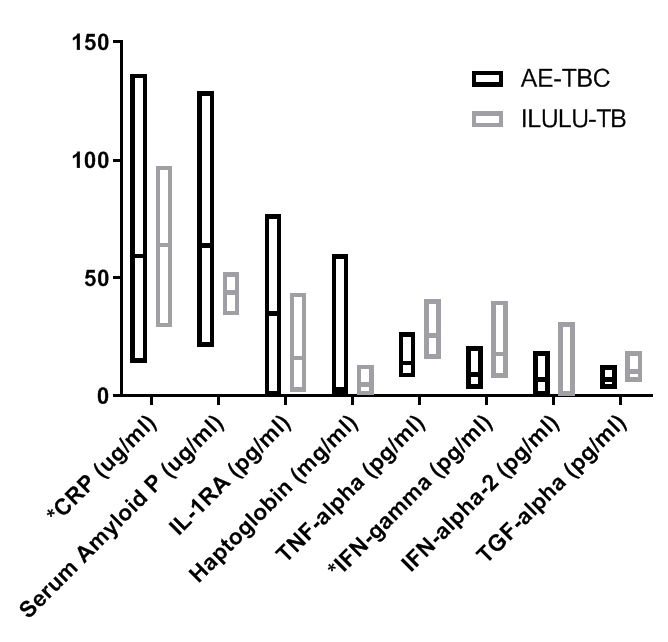 |
| --- | --- |
| 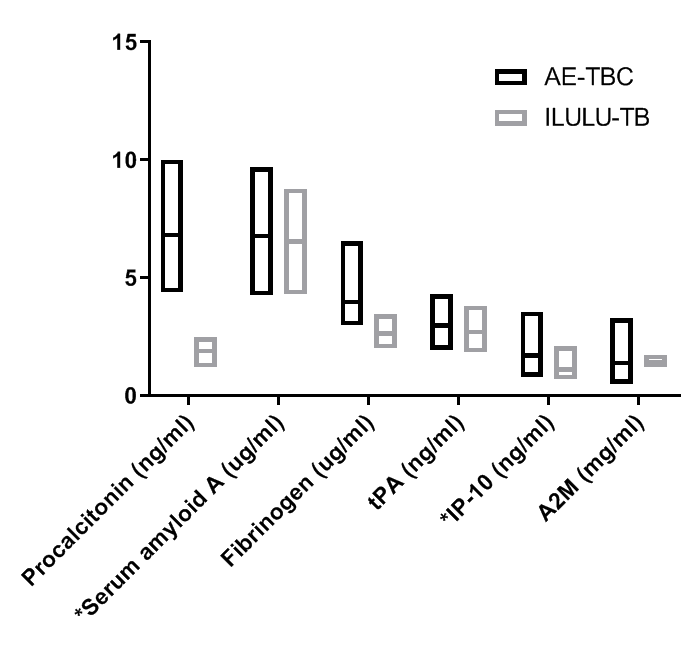 | |
